# Edge reactivity and water-assisted dissociation on cobalt oxide nanoislands

**DOI:** 10.1038/ncomms14169

**Published:** 2017-01-30

**Authors:** J. Fester, M. García-Melchor, A. S. Walton, M. Bajdich, Z. Li, L. Lammich, A. Vojvodic, J. V. Lauritsen

**Affiliations:** 1 Interdisciplinary Nanoscience Center, Aarhus University, DK-8000 Aarhus C, Denmark; 2 Chemical Engineering and SLAC National Accelerator Laboratory, Stanford University, Stanford, California 94025, USA; 3 School of Chemistry, Trinity College Dublin, Dublin 2, Ireland; 4 School of Chemistry, University of Manchester, Manchester M13 9PL, UK; 5 Department of Physics and Astronomy, Institute for Storage Ring Facilities, Aarhus, DK-8000 Aarhus C, Denmark; 6 Department of Chemical and Biomolecular Engineering, University of Pennsylvania, 220 South 33rd Street 311A Towne Building Philadelphia Pennsylvania, 19104, USA

## Abstract

Transition metal oxides show great promise as Earth-abundant catalysts for the oxygen evolution reaction in electrochemical water splitting. However, progress in the development of highly active oxide nanostructures is hampered by a lack of knowledge of the location and nature of the active sites. Here we show, through atom-resolved scanning tunnelling microscopy, X-ray spectroscopy and computational modelling, how hydroxyls form from water dissociation at under coordinated cobalt edge sites of cobalt oxide nanoislands. Surprisingly, we find that an additional water molecule acts to promote all the elementary steps of the dissociation process and subsequent hydrogen migration, revealing the important assisting role of a water molecule in its own dissociation process on a metal oxide. Inspired by the experimental findings, we theoretically model the oxygen evolution reaction activity of cobalt oxide nanoislands and show that the nanoparticle metal edges also display favourable adsorption energetics for water oxidation under electrochemical conditions.

## Abstract (web summary)

Earth abundant transition metal oxides show great promise as catalysts for the oxygen evolution reaction. Here, the authors reveal a self-assisted water dissociation mechanism and favourable theoretical adsorption energetics for water oxidation at the edge sites of cobalt oxide nano-islands.

Water is an almost unavoidable element in surface chemistry—in some cases essential, and in others undesirable. The nature of water interactions with metal oxides is a topic of special interest for geochemistry, corrosion, catalysis and electrochemistry^1^,^2^,^3^,^4^,^5^. In particular, the chemistry of water on oxide surfaces is fundamental to the operation of electrolyzers that drive the electrochemical splitting of water into hydrogen and oxygen. A particular bottleneck for the development of new renewable energy technologies based on this process is the oxygen evolution reaction (OER)^6^—one of the electrochemical half reactions for water splitting—where the current catalysts of choice are made from oxides of expensive noble metals, unfeasible for large-scale application. However, recent progress has shown that inexpensive oxides based on 3*d* transition metals are promising substitutes^7^,^8^,^9^,^10^,^11^,^12^,^13^, and detailed theoretical predictions are available for many active oxide phases^14^,^15^,^16^. Not all sites of an oxide nanoparticle are equally active, and hence current efforts are being directed towards optimizing activity empirically through nanostructuring^17^,^18^,^19^, or via enhancement by contact synergy with noble metals such as Au^8^,^20^. In particular, exfoliated layered double hydroxides have emerged as potential Earth-abundant catalysts for OER, especially cobalt oxy-hydroxide, β-CoOOH (refs 21, 22, 23), and analogous compounds from Ni and Fe. The β-CoOOH active phase adopts a two-dimensional sheet structure in which octahedral Co is sandwiched between O layers. This structure was recently suggested through indirect evidence to host the active sites for the OER on the edges of the β-CoOOH layers^19^. Other studies have considered basal plane O vacancies and under coordinated Co atoms as the active sites^24^. However, the precise nature and location of the active sites is still under debate.

In this article, we investigate the mechanism of hydroxylation resulting from water dissociation on cobalt oxide nanoislands synthesized on a single gold (111) crystal^25^, exposing both basal plane and edge sites on the island perimeter, which structurally resemble those in a β-CoOOH layer. We find that the only sites capable of dissociating H_2_O in our experiments are located on the particle edges. These results highlight a special nature and reactivity of edge sites and propose that such minority sites should be payed special attention and considered in the efforts to further understand the detailed OER pathway on oxides. Inspired by our experimental study that emphasize significant differences between edge and basal plane sites, we extend the studies by means of density functional theory (DFT) calculations on the adsorption energetics of OER intermediates to address the potential of layered cobalt oxide structures as OER catalysts. The calculations reveal very favourable properties and a low theoretical overpotential for cobalt terminated edges, that is, the same sites also responsible for the observed water dissociation. Our theoretical modelling of the process observed in the experiment furthermore indicates that all the steps in the dissociation process, as well as in the following hydroxyl migration, are assisted by an additional water molecule, acting to lower the energy barriers involved.

## Results

### Hydroxylation of CoO nanoislands

**Figure 1:**
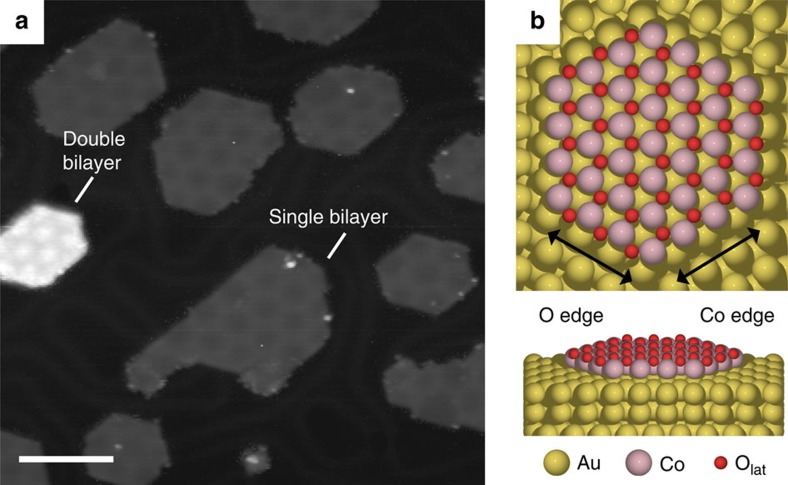
Cobalt oxide nanoislands on a Au(111) surface. (**a**) Representative STM image showing a 30% ML synthesis of CoO nanoislands. Single and double bilayers are labelled. (**b**) Ball structure model showing a pristine hexagonal single bilayer island expressing both cobalt and oxygen edges. Scale bar: (**a**) 100 Å.

The majority (95%) of the cobalt oxide nanoislands synthesized on Au(111) are single Co–O bilayers that coexist with a minority (5%) of double Co–O bilayers, both oriented with a pristine close-packed O-terminated (111) basal plane parallel to the substrate with a cobalt layer in direct contact with the gold support (Fig. 1). These structures and their relative abundance remain essentially unchanged from low coverage (∼10%) to the monolayer limit (∼95%). The predominant hexagonal shape of the nanoislands dictates two different low-index edge structures terminating the islands, one exposing a row of cobalt atoms (Co edge), and the other a row of oxygen atoms (O edge) (Fig. 1b). The edge structure of the double bilayers, which adopts the wurtzite structure, is more complicated and expresses both of the cobalt and oxygen terminations.

**Figure 2:**
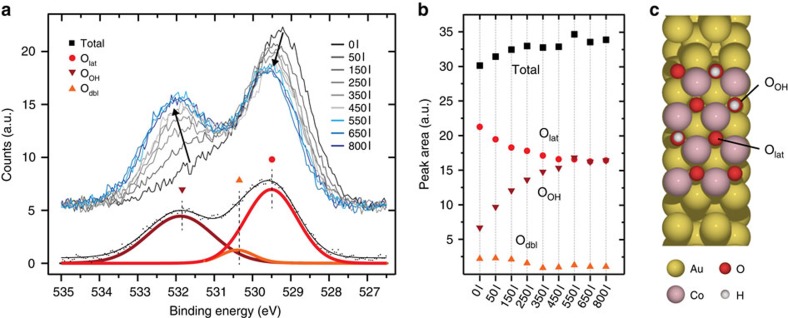
Hydroxylation of CoO nanoislands monitored by XPS. (**a**,**b**) XPS spectra (*hν*=625 eV) and individual peak areas of the O 1*s* core level during water exposure at 2 × 10^−7^ mbar. (**c**) Ball model showing the individual oxygen species O_OH_ and O_lat_.

To study water dissociation, we exposed the cobalt oxide nanoislands to water vapour (*p*(H_2_O)=2 × 10^−7^ mbar) at room temperature and investigated the surface with X-ray photoelectron spectroscopy (XPS). Figure 2a shows the O 1*s* XPS spectrum of the islands as a function of water exposure measured in Langmuir (denoted by the unit L, where 1 L=1 × 10^−6^ torr for 1second). The O 1*s* spectrum radically changes as a function of exposure. This can be monitored in more detail by deconvolution of the spectra into individual peaks, corresponding to distinct oxygen species. We find that three peaks are required to produce a satisfactory fit for the entire series. The low binding energy (BE) peak is predominant in the spectrum recorded for pristine nanoislands and represents lattice oxygen (O_lat_) within the nanoislands. The higher binding energy peak at 532.0 eV, on the other hand, increases significantly with water exposure and can be assigned to hydroxyl groups (O_OH_)^2^. This hydroxylation results from water dissociation on the nanoislands and causes a corresponding drop in O_lat_ as a function of water exposure, indicating that lattice oxygen atoms are hydroxylated in the process (Fig. 2b). At high water exposures (>600 l), no further change is observed, thus implying that saturation hydroxylation has been reached with a ratio of O_lat_ to O_OH_ of 1:1. We ascribe the weak intermediate energy peak to buried lattice oxygen present in the double bilayer islands (O_dbl_). The peak's intensity does not show dramatic changes throughout the experiment and the O_dbl_ do not appear accessible for hydroxylation under our experimental conditions.

**Figure 3:**
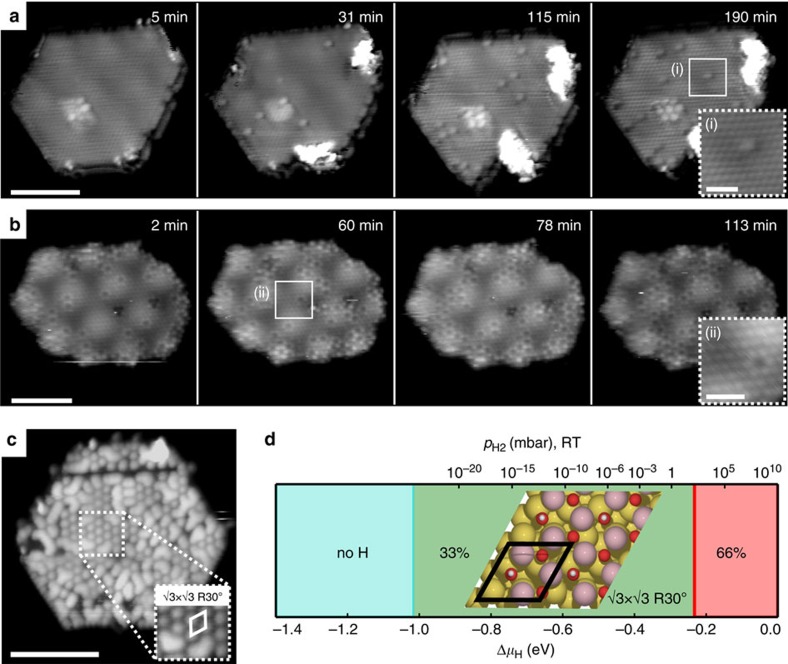
*In situ* STM movies and saturation level of hydroxylation. (**a**,**b**) Image sequences from atom-resolved STM movie (supplementary) recorded during water exposure showing the initial stage of hydroxylation on single- (**a**) and double- (**b**) bilayer CoO nanoislands. The insets show high magnification atom-resolved STM images of hydroxyl groups on a single- (i) and double-bilayer (ii). (**c**) STM image of a heavily hydroxylated single bilayer island with areas of ordered 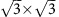
R30° superstructure. (**d**) DFT-calculated hydroxylation structure of the CoO basal plane as a function of H chemical potential and pressure at room temperature (upper scale). Scale bars: (**a**) 50 Å (inset 12 Å), (**b**) 50 Å (inset 6 Å), (**c**) 50 Å.

The progress of hydroxylation of the islands was investigated in parallel in atom-resolved scanning tunnelling microscopy (STM)^26^ movies by dosing water into the chamber *in situ* during continuous STM scanning at room temperature (Supplementary Movie 1 (single bilayer) and 2 (double bilayer)). Point-like features start to appear on the basal planes of both island types (Fig. 3a,b) with a density directly correlated with water exposure. On both structures they occupy O_lat_ positions and are assigned to hydroxylated oxygen atoms, that is, H adsorbed on O_lat_ sites. STM shows that hydroxyl groups are present in low numbers on even pristine islands due to residual water vapour in the rest gas, which is in agreement with the small tail seen in the XPS data at 0 l exposure (Fig. 2a). Hydroxyl groups on single bilayer islands usually appear as faint protrusions (Fig. 3a, inset i), while appearing as ‘donut'-shaped depressions on double bilayer islands (Fig. 3b, inset ii). A variety of other imaging modes are also possible (Supplementary Fig. 1) since the STM contrast on oxides is in general highly sensitive to the STM tip termination^27^.

STM images recorded on syntheses exposed to high water dosages further than that shown in the STM movies (>1,000 l) display the emergence of an ordered 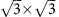
R30° superstructure (with respect to the O_lat_ positions) of hydroxyls (Fig. 3c). In agreement, our density functional theory calculations within the Hubbard U approximation (DFT+U) show that the expected optimal OH coverage (*θ*_OH_, defined as the ratio of hydroxyls and number of basal plane O atoms) under the experimental conditions for the defect-free basal plane is *θ*_OH_=0.33, reflected by such a 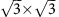
R30° overlayer structure of the adsorbed H (Fig. 3d). However, coexisting with the 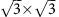
R30° superstructure are linear chains of hydroxyls which interconnect between domains, meaning the overall hydroxylation measured is closer to *θ*_OH_≈0.5, as observed by XPS measurements (Fig. 2b). This is in contrast to the similar system of supported thin films of FeO, for which our calculations find an optimal *θ*_OH_ of 6% when supported on Au (Supplementary Fig. 2), consistent with previous experimental observations^28^. The tendency to form the 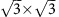
R30° superstructure is indicative of hydroxyl–hydroxyl repulsion, a phenomenon that has been also observed on other oxide surfaces^1^,^29^.

### Reaction mechanism of water dissociation on CoO edge sites

**Figure 4:**
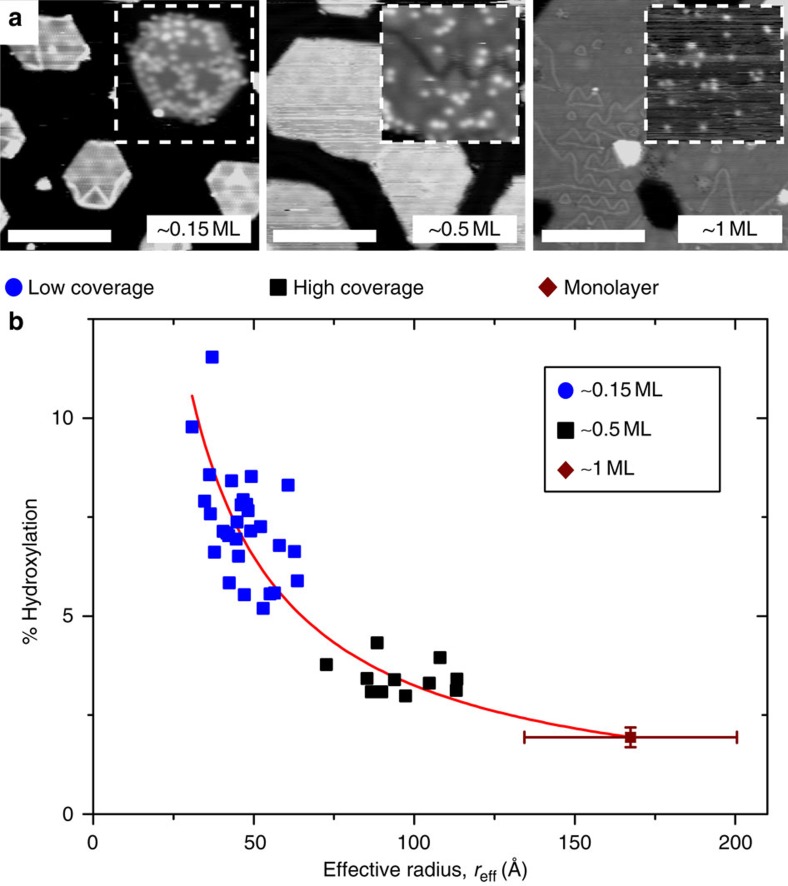
Relationship between edge length and activity. (**a**) STM images from a coverage series varying the amount of deposited and oxidized cobalt. Insets (100 × 100 Å^2^) show sample areas after exposure to 200 l H_2_O. (**b**) Effective island radius versus degree of hydroxylation after 200 l H_2_O estimated from atom-resolved STM images for different sized nanoislands showing an inversely proportional relationship. Note that the degree of hydroxylation was kept far below saturation (50%) to facilitate the image acquisition and counting of individual hydroxyls. The error bars indicate the s.d. on individual measurements of the hydroxylation and effective radius. Scale bars: (**a**) 75 Å.

The atom-resolved STM movies also reveal that the new hydroxyls originate from the edges of the islands before they diffuse to the basal plane (compare for example, panels 1 and 2 of Fig. 3b). This clearly suggests that island edges are the active sites for water dissociation and that hydroxyl groups diffuse from these sites to the centre of the island. To prove this, three different syntheses were made with different edge/basal plane ratios by varying the cobalt oxide island coverage from ∼10% (yielding small islands) to an almost complete monolayer CoO film (Fig. 4). These syntheses were exposed to the same total dosage of 200 l water, which is high enough to cause measureable hydroxylation in atom-resolved STM, but far from saturation. The surface hydroxyl coverage *θ*_OH_ was measured and compared with the effective island radius (*r*_eff_, proportional to the inverse of edge to basal plane ratio) for a number of islands in STM images in each synthesis. The hydroxyl density *θ*_OH_ follows exactly a trend inversely proportional to the radius, in agreement with edge activity, as dissociation on the basal plane sites would result in a constant *θ*_OH_ independent of *r*.

**Figure 5:**
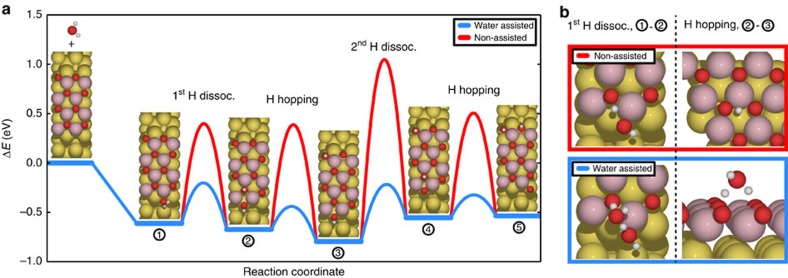
Comparison of water assisted and non-assisted water dissociation mechanisms. (**a**) Reaction intermediates are: (1) water adsorption, (2) first H dissociation onto a basal plane oxygen, (3) hopping of the first proton to the basal plane. Note the H ends up on the O-edge due to its close vicinity. (4) Second hydrogen dissociation onto a basal plane oxygen, and (5) hopping of the second proton. (**b**) Assisted- and non-assisted reaction transition states in 1st H dissociation and H hopping. Colour code Co: light red, O: dark red, Au: yellow.

Our DFT+U simulations on a series of systems consisting of two-dimensional periodic cobalt oxide stripes on a Au(111) surface (see Methods section^30^,^31^) reveal a favourable reaction pathway for water dissociation on the edges. Although these modelled stripes expose both the O-terminated basal plane together with Co- and O-terminated edges on opposite sides (Fig. 1b), no favourable reactions were found on the O edge, nor on the O-terminated basal plane of island. The lowest energy diagram obtained for the dissociation of a single water molecule on the Co edge is presented in Fig. 5a. This process starts with the adsorption of the water molecule to a Co-site in an on-top configuration (1) followed by the first H-dissociation, in which one hydrogen atom is transferred to the nearest neighbour oxygen on the basal plane. For this process, we find a relatively high activation barrier (*E*_a_=1.01 eV), which is at first inspection inconsistent with the reactivity observed in the experiments at room temperature. Therefore, taking into account the water vapour background during the hydroxylation process, this step was modelled again in the presence of another water molecule in the vicinity. The influence of the additional water molecule is surprisingly strong, finding a significantly lower barrier of 0.41 eV. This demonstrates that the additional water assists water dissociation on the exposed Co sites by acting as a proton shuttle in form of a H_3_O species in the transition state (Fig. 5b). Such process resembles the autocatalytic water dissociation reported for bare metal surfaces^32^,^33^ and is the direct evidence that a water assisted dissociation process may proceed at under coordinated sites on a metal oxide.

The hydrogen originating from the dissociated water in configuration (2) subsequently diffuses away from the Co-metal edge to energetically preferable configurations on the basal plane O lattice of the cobalt oxide stripe as in experiment (and eventually onto the nearby O edge in the calculation (3)). Again, we find that the associated hydrogen hopping is much more efficient when it is assisted by water (*E*_a_=0.23 eV), as opposed to the non-assisted hopping (*E*_a_=1.06 eV), similar to a previous study on H diffusion a FeO thin film^34^. The fundamental effect of the reduction in the reaction barrier relates to the formation of H_3_O species in the transition state formed from the water and the hydrogen on the surface. Upon dissociation of the H_3_O species, the excess H adsorbs again on the surface with equal probability on all three neighbouring O sites leading to overall diffusion of H across the surface. In the STM movies presented in Fig. 3a,b, the hydrogen mobility is clearly evident due to the background water pressure during the acquisition of both movies. Contrary, at the base UHV pressure (10^−10^ mbar), hydroxyls are almost completely static, as expected from the calculations. Moreover, the mobility was directly shown to strongly correlate with water vapour pressure by recording an STM movie similar to Fig. 3a,b, but varying the water pressure *in situ* (Supplementary Fig. 3).

The second dehydrogenation of the OH intermediate on the Co edge (steps 3–4 in Fig. 5) is slightly higher in energy (*E*_a_=0.57 eV) compared with the first H dissociation step, but again strongly water assisted, following the same mechanism. The water dissociation process is −0.54 eV downhill in total energy at 0 K, while energy neutral at typical conditions of the experiment. Overall, the calculations indicate that water plays a far greater role than previously thought, not only assisting hydrogen hopping, but also mediating all the elementary steps of its own dissociation.

The final state of the water dissociation process results in O adsorbed on the Co edge with H atoms spatially separated from the edge onto positions on the basal plane, as observed in the STM experiments. Further DFT+U simulations reveal that dissociation of additional water is not likely at room temperature when all Co edge sites are O-covered (Supplementary Figs 4 and 5), resulting in a maximum theoretical hydroxyl density of *θ*_OH_=*2n*_edge_*/n*_basal_, where *n*_edge_ and *n*_basal_ are the number of sites on the island edge and basal plane, respectively. In a typical 30% ML synthesis (Fig. 1a), the measured ratio of edge atoms to basal plane atoms (*n*_edge_*/n*_basal_) is between 0.10 and 0.15 giving an expected maximum hydroxylation of *θ*_OH_=0.20–0.30. Surprisingly, we always observe a significantly higher hydroxylation of *θ*_OH_ ≈ 0.5 in both XPS and STM at saturation (Figs 2b and 3c). This excess hydroxylation in violation of the limit set by the available edge sites may be due to a number of contributing factors. Firstly, the ratio of edge atoms to basal plane atoms for a given island is calculated assuming perfect edges containing no kinks or single atom vacancies. However, such features can be observed at many island edges (Fig. 1a). Secondly, STM movies recorded during water exposure reveal that edge structures are highly dynamic upon water exposure with constant restructuring occurring (Supplementary Fig. 6). This suggests that fresh Co sites might be exposed dynamically even at room temperature. We rule out that such processes could be induced by the vicinity of the tip since independent XPS data shows the same degree of saturation. Therefore, we propose that dynamic rearrangements of the CoO edge structure might be generally occurring at room temperature and important for the dissociative properties of the islands.

Our experimental results of the model system directly reveal that exposed Co atoms act to dissociate water on the cobalt oxide nanoparticle edges, resulting in a hydroxylation of the oxide. Inspired by these findings based on studies under vacuum conditions, we have further investigated the energetics for electrochemical water oxidation on the cobalt oxide particle edges versus basal plane sites by means of DFT, addressing the existing hypothesis that the activity of layered cobalt oxyhydroxide nanocatalysts (strongly resembling our model structure) is related to the edge length^19^.

The conventional OER mechanism in CoO_*x*_ and NiO_*x*_ catalysts in alkaline media is based on a single-site OH^−^/e^−^ exchange mechanism^16^,^35^,^36^,^37^,^38^, although alternative mechanisms involving superoxo-type species have been also proposed^7^,^35^,^39^. Regardless of the type of mechanism, specific sites with favourable energetics for the O and OH intermediates need to exist for efficient OER to occur. We have modelled the reactivity under OER conditions of all the possible sites in gold supported Co–O and the related O–Co–O trilayer islands^22^ using the conventional OER mechanism (Table 1). For a better comparison with previous OER studies on Co-oxides^16^,^36^, these energies have been calculated at the PBE+U (*U*=3.32 eV) level. We find that all of these steps require the interaction of the OER intermediates (that is, OH*, O* and OOH*) at the under coordinated Co site at edges of the bilayer and trilayer nanoislands. Additionally, we have calculated the formation energies of vacancies on the fully O saturated basal plane, O edge and Co edge sites. Here, the O sites at the basal plane stand out as the sites with the highest oxygen vacancy formation energy (Supplementary Fig. 4). This finding is in agreement with the experiment, where we never observe O vacancies on the basal plane. Furthermore, the calculated energetics of the OH* and O* intermediates at the basal plane of both bilayers and trilayers are clearly outside the optimum range defined by a theoretical overpotential below 1 V (refs 14, 16, 37, 38). Therefore, calculations predict a very unfavourable OER activity for these sites (Table 1). This leaves the exposed Co edges as the only sites likely to dissociate water and act as OER active sites, with a calculated overpotential of only 330 mV for the O–Co–O trilayers.

The water dissociation, as studied in our experimental work, may not be the overall rate-limiting step in the OER mechanism, and in alkaline media the OER pathway does not necessarily involve the dissociation of water since OH is readily available from the solution. Furthermore, under biased electrochemical conditions the possibility exists that the dissociation at edge sites occurs more facile elsewhere on the cobalt oxide due to changes in the chemical state of the cobalt. However, our experimental study shows that the interactions between oxide and water on edges are remarkably different from basal plane sites for simple reactions such as the dissociation process. We propose that these sites might in general play a significant role in the water oxidation pathway, as well as other reactions. In addition, our calculations applicable to biased electrochemical conditions (using trilayer islands), again pinpoint the edges as the preferable active site for the OER, and thus support the recent indirect evidence that β-CoOOH layers host the active sites for the OER on the edges^19^. To confirm these findings, the well-defined number and controlled variation of edge sites in the model catalyst may be used to quantify edge reactivity in a working model electrode setup under electrochemical conditions. Lastly, the microscopic picture of water dissociation and water-assisted hydrogen mobility on cobalt oxide revealed in this work is also important for the general understanding and further modelling of the emerging class of Earth-abundant metal oxide catalysts for the OER, as well as other relevant catalytic reactions in aqueous media.

## Methods

### Experimental methods

All syntheses and analysis was performed in UHV chambers with a base pressure of ∼1 × 10^−10^ mbar. Cobalt oxide nanoislands were synthesized on a clean and flat Au(111) surface using the synthesis described in (ref. 25). Briefly, this consists of evaporation of cobalt from an e-beam evaporator in an oxygen environment (1 × 10^−6^ mbar O_2_) and a post-annealing step at 523 K in the same environment and a final brief anneal at 523 K in UHV conditions. Milli-Q grade water was dosed from a glass vial into the UHV chamber via a leak valve. The water was first degassed by repeated freeze–pump–thaw cycles. The resultant islands were imaged using an ‘Aarhus'-type STM and XPS was done using the Matline endstation on the ASTRID II synchrotron, Aarhus (operating in ‘top up' mode). O 1*s* spectra were recorded at normal emission and a photon energy of 625 eV. The spectra were energy calibrated using the Au 4*d* 5/2 peak at a binding energy of 335.0 eV as a reference. There was no X-ray beam present on the sample during the water dosing.

### Computational methods

DFT calculations were performed using the projector augmented wave (PAW) potentials^40^ within the Vienna *ab initio* simulation package^41^,^42^ VASP, version 5.3.5). As in the study of CoO on Ir(100)^43^, FeO on Pt(111)^1^,^2^, and also in our recent study of CoO nanoislands^25^, we adapted the PBE^44^ functional together with the Hubbard-*U* method^45^ with a value of *U*_eff_ =1 eV applied for the *d*-electrons of Co atoms. A standard energy cutoff of 400 eV and an approximately constant *k*-point mesh of 8 × 8 × 1 points per *p*(1 × 1) unit cell was kept in all slab calculations. In all cases, a metal-oxide bilayer and the upper most top layer of Au(111) were allowed to relax with the maximum force threshold of 0.02 eV Å^−1^, while at least 11 Å of vacuum was used to separate the layers. The magnetic structure was always taken to be the row-wise AFM structure, RW-AF-(2 × 1), known from FeO bilayers^46^.

The hydroxylation of basal planes was studied using infinite surfaces of periodicities ranging from *p*(1 × 1), *p*(✓3 × ✓3), *p*(2 × 2), *p*(3 × 3) to *p*(4 × 4) with the metal-oxide layer placed in a FCC stacking relative to a commensurate Au(111) lattice (Supplementary Fig. 2). One exception from this rule was the case of *p*(✓3 × ✓3) metal-oxide, where we also adopted incommensurate *p*(✓3 × ✓3)/Au(2 × 2) structures known from a previous work on FeO on Pt(111)^47^. To study the energetics of the edges of the nanoislands, we used stripes of periodicity *p*(2 × 4) CoO FCC stacked within the *p*(2 × 8) unit cell of Au(111), referred to as *p*(2 × 4/2 × 8). Size effects were also tested for some reaction intermediates using two times wider *p*(4 × 4/4 × 8) stripes leading to very similar results. The transition states for water dissociation and hydrogen hopping were located by means of the climbing image nudged elastic band (CI-NEB) method, using at least five images along the reaction coordinate^45^. The nature of the transition states was assessed by performing numerical frequency analysis to confirm the existence of only one imaginary vibrational mode.

### Data availability

The data that support the findings of this study are available from the corresponding author upon reasonable request.

## Additional information

**How to cite this article:** Fester, J. *et al*. Edge reactivity and water-assisted dissociation on cobalt oxide nanoislands. *Nat. Commun.* **8,** 14169 doi: 10.1038/ncomms14169 (2017).

**Publisher's note**: Springer Nature remains neutral with regard to jurisdictional claims in published maps and institutional affiliations.

## Supplementary Material

Supplementary Information Supplementary Figures

Supplementary Movie 1 "STM movie (155Åx135Å, I=-0.28nA and V=-911.2mV) of a single bilayer CoO island during in-situ exposure to 2×10^-7^ mbar H_2_O at room temperature. Time difference between single images: 82.02s."

Supplementary Movie 2 STM movie (150Åx120Å, I=-0.26nA and V=-806.0mV) of a double bilayer CoO island during in-situ exposure to 5×10^-8^ mbar H_2_O at room temperature. Time difference between single images: 36.7s."

## Figures and Tables

**Table 1:** Calculated OER energetics at the available basal plane sites and edge sites of the bilayer (Co–O) and trilayer (O–Co–O) nanostripes.

**Specific site/OER energetics**	**Δ*****G***_OH*_(eV)	**Δ*****G***_O*_(eV)	**Δ*****G***_OOH*_(eV)	**Calculated overpotential** ***η***_**cal**_ **(V)**
OER active range (min, max)	(−0.5, 2.2)	(1.7, 4.4)	(2.7, 5.4)	<1
*Basal plane OER energetics*				
Bilayers: OH-site on basal plane	−0.57	0.05	NA	>1 (ads. too strong)
Bilayers: O-site on basal plane	−0.48	−0.02	NA	>1 (ads. too strong)
Trilayers: OH-site on basal plane	−1.09	0.06	NA	>1 (ads. too strong)
Trilayers: O-site on basal plane	−1.39	−0.77	NA	>1 (ads. too strong)
				
*Edge-site OER energetics*				
Bilayers: top of the metal-edge	1.37	3.03	4.53	0.43
Trilayers: bottom of O-edge near Au	0.17	1.99	3.31	0.60
Trilayers: top of the metal-edge	1.51	3.03	4.60	0.33 (optimal)
