# Supplementary material for: Edge reactivity and water-assisted dissociation on cobalt oxide nanoislands
Source: Nat Commun. 2017 Jan 30;8:14169. doi: 10.1038/ncomms14169 (PMC5290272; doi:10.1038/ncomms14169)
Supplement: Supplementary Information — Supplementary Figures [file ncomms14169-s1.pdf]

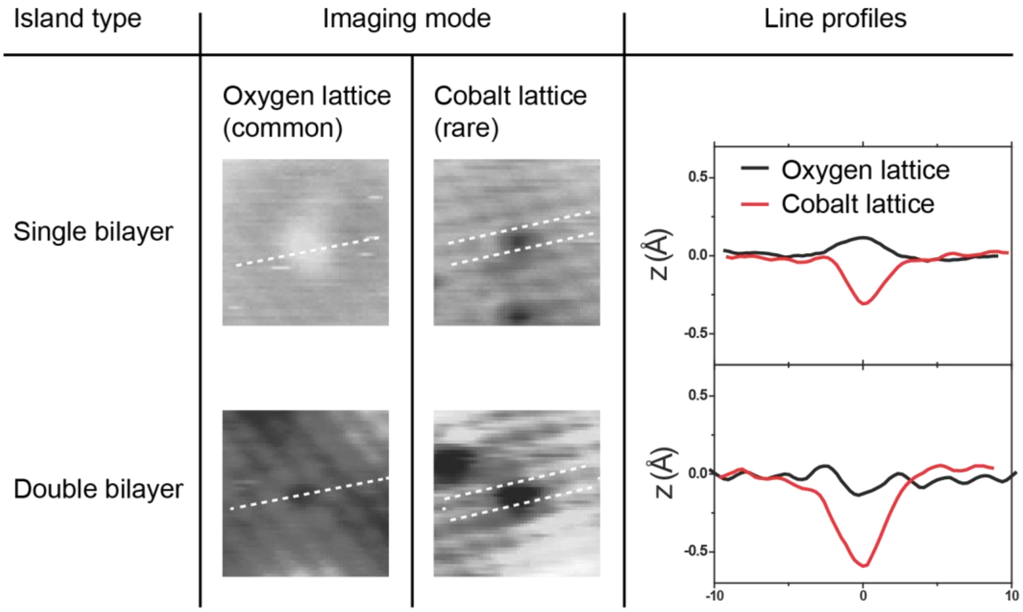

Supplementary Figure 1

Hydroxyls on the basal plane of cobalt oxide nanoislands imaged by atom-resolved STM. Various imaging modes lead to different appearances and hydroxyls appear different on single- and double bilayer islands. The most common imaging mode (~95%) shows the hydroxyls as a faint protrusion on single bilayer islands and as a “donut-like” depression on double bilayer (left column). Both features are in registry with the imaged atomic lattice and the imaging mode is as such referred to as the “oxygen mode”, displaying the top O_{lat} oxygen atoms. The rarer imaging mode (right column) shows the hydroxyls as deep depressions on both single and double bilayer islands (second column). These are out of registry with the surface atoms and the imaging mode is referred to as the “cobalt mode” (the STM imaging the underlying cobalt atoms).

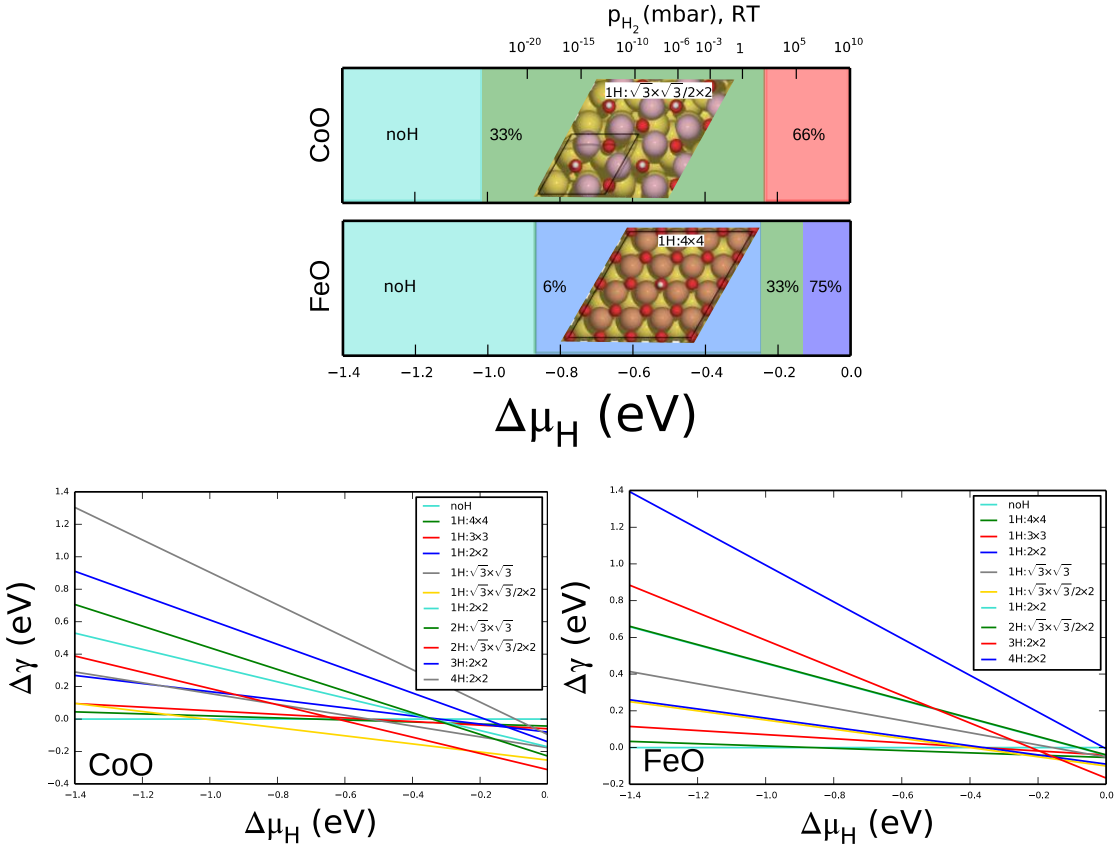

Supplementary Figure 2

Top: Calculated hydroxylation levels of the CoO and FeO basal planes as function of hydrogen chemical potential at room temperature (RT) using the surface energies Δγ of infinite films. Bottom: Surface energies Δγ of CoO (left) and FeO (right) infinite films with various periodicities as mentioned above.

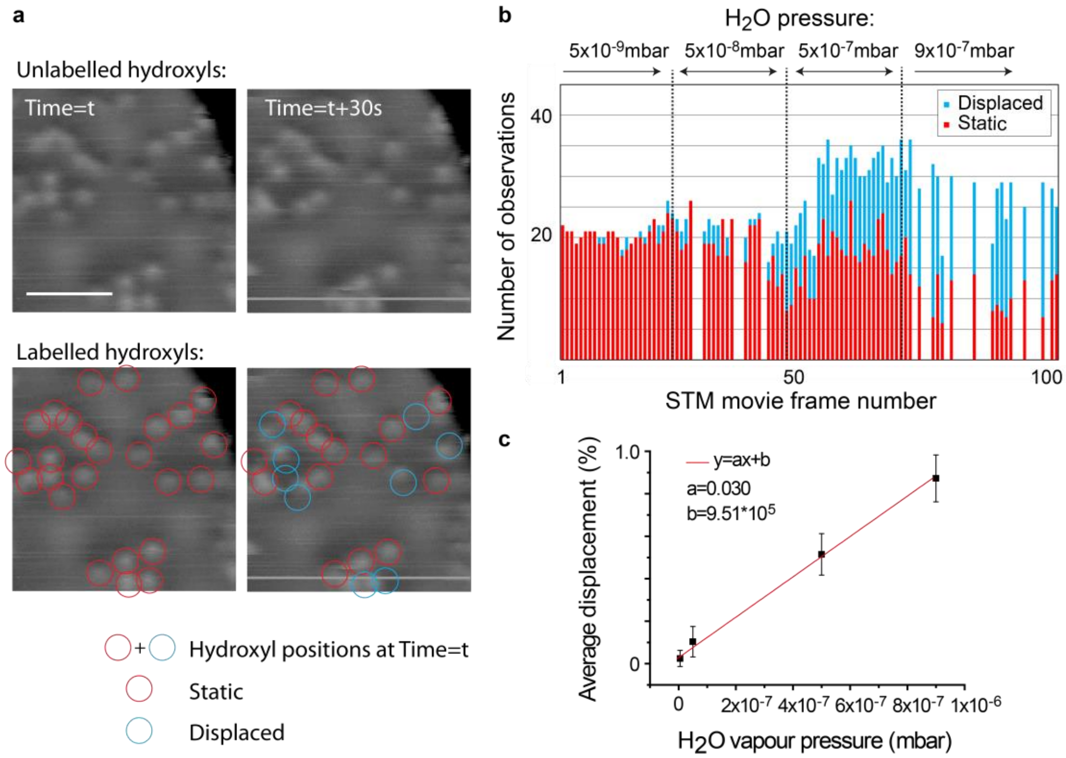

Supplementary Figure 3

Hydroxyl mobility analysis of a CoO single bilayer island from an STM movie. **a**, Example of determination of a degree of mobility defined as the percent of hydroxyls displaced (marked by blue rings) since last frame. Note that this analysis does not take into account the distance moved. **b,** Statistics of the number of displaced versus static hydroxyls between every pair of consecutive frames during the whole STM movie. Only hydroxyls well defined in both consecutive STM images were taken into account. Step changes in the H_2O pressure are indicated by vertical lines. **c**, Average displacement percent in the four H_2O pressure regions. These averages were corrected, taking into account the possibility of multiple movements of the same hydroxyl, assuming the number of displacement events follow a poisson distribution. The errorbars indicate the

standard deviation on the average of the displacement percent between consecutive images within each H_2O pressure region. The STM imaging conditions throughout the movie were I=-0.37nA and V=-1277.1mV (30.05s per frame). Scale bar: a) 25Å.

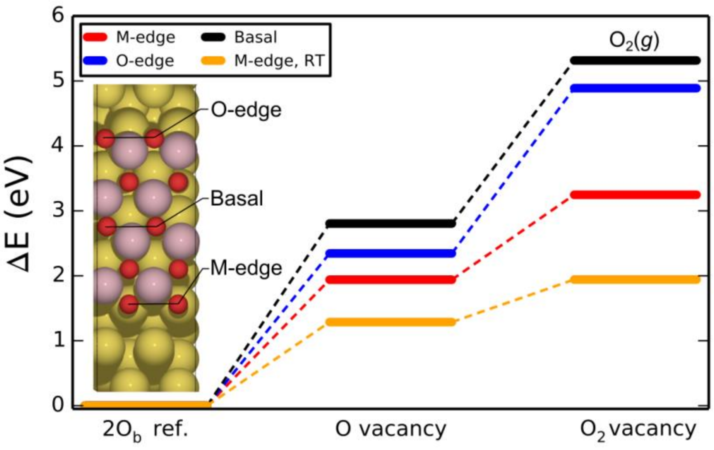

Supplementary Figure 4

Calculated relative stability of three distinct oxygen sites at the metal-edge (red line), oxygen-edge (blue line) and basal plane (black line) in a CoO nanoisland modeled in the form of 2×4 stripe. When referencing the oxygen chemical potential to the O_2 molecule in the gas phase, all three sites show high level of thermodynamic stability. As expected, the oxygen at M-edge site is the easiest to remove. At room temperature (RT), this process amounts to be about 2 eV uphill in energy.

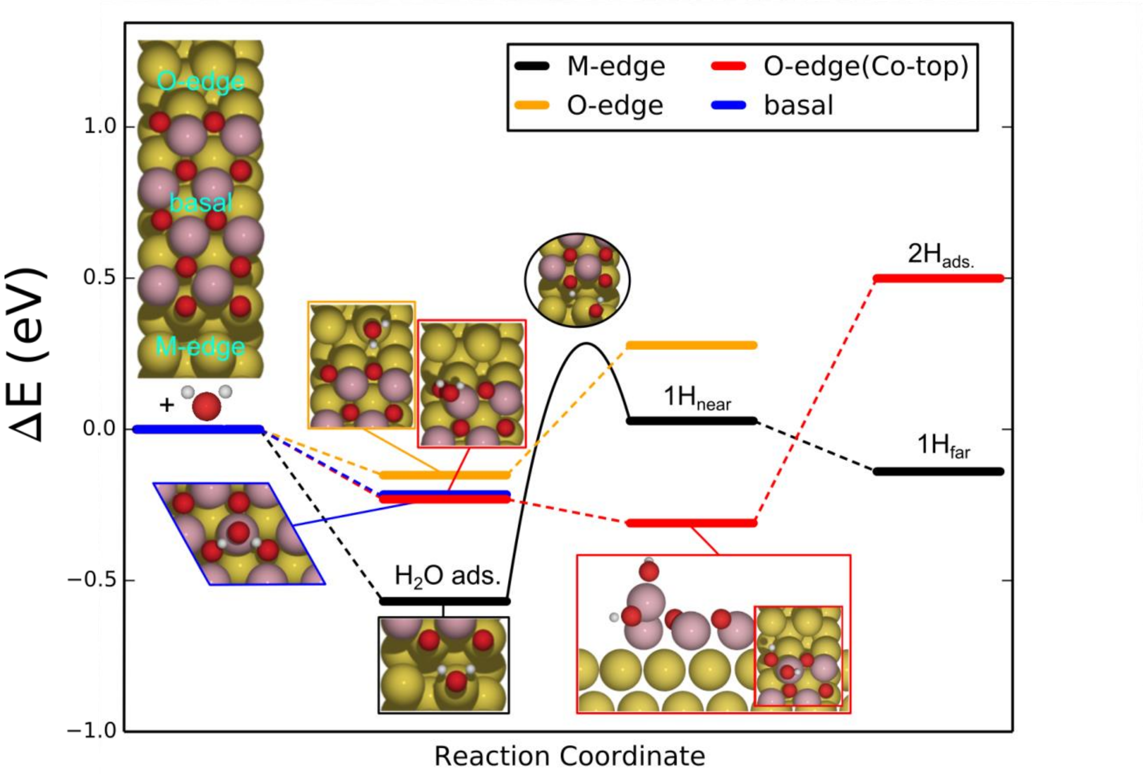

Supplementary Figure 5

Comparison of additional water adsorption and dissociation pathways. The energetics of water adsorption (H_2O ads.) and proton transfer steps ($1H_{near/far}$, $2H_{ads.}$) is calculated for the oxidized Co-edge site (black line), O-edge site (red and orange lines) of a 2×4 stripe and for the basal plane site (blue line). Only the O-edge site (red line) with an out-of-plane Co-metal site has favorable energetics towards the first hydrogen transfer from water to the stripe. The activity of such distorted geometry suggests the importance of defects for the water dissociation process.

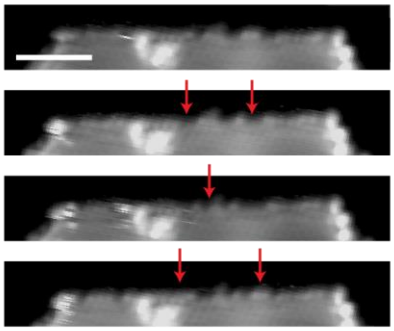

Supplementary Figure 6

Sequential frames from an STM movie (I=-0.30nA and V=-875.8mV, Δt = 60s) showing edge dynamics during water exposure at p=2.5×10^{-8} mbar H_2O. Arrows indicate the most significant differences between frames. Scale bar: 25Å.
